# Supplementary material for: Active poly‐GA vaccination prevents microglia activation and motor deficits in a C9orf72 mouse model
Source: EMBO Mol Med. 2019 Dec 20;12(2):e10919. doi: 10.15252/emmm.201910919 (PMC7005532; doi:10.15252/emmm.201910919)
Supplement: Supplementary file 1 — Appendix [file EMMM-12-e10919-s001.pdf]

Active poly-GA vaccination prevents microglia activation and motor deficits in a *C9orf72* mouse model

Qihui Zhou, Nikola Mareljic, Meike Michaelsen, Samira Parhizkar, Steffanie Heindl, Brigitte Nuscher, Daniel Farny, Mareike Czuppa, Carina Schludi, Alexander Graf, Stefan Krebs, Helmut Blum, Regina Feederle, Stefan Roth, Christian Haass, Thomas Arzberger, Arthur Liesz, Dieter Edbauer

Running title: Poly-GA vaccination in C9orf72 ALS/FTD

Key words: ALS, FTD, *C9orf72,* neurodegeneration, immunotherapy

Table of contents

Supplemental Tables

Table S1: Statistical analysis for anti-GA response in Fig. 1B

Table S2: Statistical analysis for beam walk test in Fig. 2A

Supplemental Tables

Table S1

Statistical analysis for anti-GA response in Fig. 1B

Age (weeks)	Group	P value
7	TG-PBS vs WT-PBS	>0.9999
	TG-Ova-$(GA)_{10}$ vs WT-PBS	>0.9999
	TG-Ova-$(GA)_{10}$ vs TG-PBS	>0.9999
9	TG-PBS vs WT-PBS	>0.9999
	TG-Ova-$(GA)_{10}$ vs WT-PBS	>0.9999
	TG-Ova-$(GA)_{10}$ vs TG-PBS	>0.9999
13	TG-PBS vs WT-PBS	>0.9999
	TG-Ova-$(GA)_{10}$ vs WT-PBS	0.8286
	TG-Ova-$(GA)_{10}$ vs TG-PBS	0.8464
17	TG-PBS vs WT-PBS	>0.9999
	TG-Ova-$(GA)_{10}$ vs WT-PBS	<0.0001
	TG-Ova-$(GA)_{10}$ vs TG-PBS	<0.0001
21	TG-PBS vs WT-PBS	>0.9999
	TG-Ova-$(GA)_{10}$ vs WT-PBS	<0.0001
	TG-Ova-$(GA)_{10}$ vs TG-PBS	<0.0001
25	TG-PBS vs WT-PBS	>0.9999
	TG-Ova-$(GA)_{10}$ vs WT-PBS	<0.0001
	TG-Ova-$(GA)_{10}$ vs TG-PBS	<0.0001
29	TG-PBS vs WT-PBS	>0.9999
	TG-Ova-$(GA)_{10}$ vs WT-PBS	<0.0001
	TG-Ova-$(GA)_{10}$ vs TG-PBS	<0.0001

Table S2

Statistical analysis for beam walk test in Fig. 2A

	TG-PBS vs WT-PBS	TG-OVA-$(GA)_{10}$ vs WT-PBS	TG OVA-$(GA)_{10}$ vs TG-PBS	TG-$(GA)_{15}$ vs WT-PBS
9 weeks	0.8730014	0.914881408	0.660597254	0.282132
11 weeks	0.544982788	0.544982788	0.971550741	0.607581
13 weeks	0.412916226	0.412916226	0.711801435	0.412916
15 weeks	0.010732991	0.043912676	0.297386709	0.041121
17 weeks	0.00405365	0.018344738	0.164924499	0.010073
19 weeks	0.003147226	0.481335516	0.021661226	0.002403
21 weeks	0.001395696	0.032603915	0.029964124	0.001396
23 weeks	0.005474251	0.008110434	0.103991363	0.00811
25 weeks	0.003995691	0.066337767	0.073134623	0.001266
27 weeks	0.005370445	0.12551599	0.01600375	0.00537
29 weeks	0.001353034	0.022590417	0.01546881	0.001353
31 weeks	0.00170716	0.084476449	0.026083767	0.001707
