# Supplementary material for: Active poly‐GA vaccination prevents microglia activation and motor deficits in a C9orf72 mouse model
Source: EMBO Mol Med. 2019 Dec 20;12(2):e10919. doi: 10.15252/emmm.201910919 (PMC7005532; doi:10.15252/emmm.201910919)
Supplement: Supplementary file 2 — Expanded View Figures PDF [file EMMM-12-e10919-s002.pdf]

Expanded View Figures

Figure EV1. Immunization regimen caused no overt side effects in mice.

A Body weight curve of vaccinated female mice (left panel) and male mice (right panel) on a standard diet. Number of mice per group as indicated. Two-way repeated-measure ANOVA (group × time) revealed a non-significant main effect of group (females: $F_{2,13}$ = 0.3747, P = 0.6947; males: $F_{2,11}$ = 3.042, P = 0.0888) and significant effect of time (females: $F_{5,65}$ = 13.28, $P < 0.0001$; males: $F_{5,55}$ = 102.6, $P < 0.0001$) and a non-significant interaction between factors (females: $F_{10,65}$ = 0.8671, P = 0.5678; males: $F_{10,55}$ = 1.067, P = 0.4030), followed by Tukey's *post hoc* test.

B Leukocyte distribution in the spleen measured by flow cytometry at 32 weeks of age, n = 5–7 mice per group as indicated. Gating of the different cell populations is shown in a representative scatter plot. Tukey-style box plot shows 25th, 50th, and 75th percentiles, and whiskers extend to $\pm$ 1.5 interquartile range. One-way ANOVA and Tukey's *post hoc* revealed no significant changes in the frequency of different cell subtypes. B cells: P = 0.4962; monocytes: P = 0.8126; CD4 T cells: P = 0.9443; CD8 T cells: P = 0.6648.

© 2019 The Authors

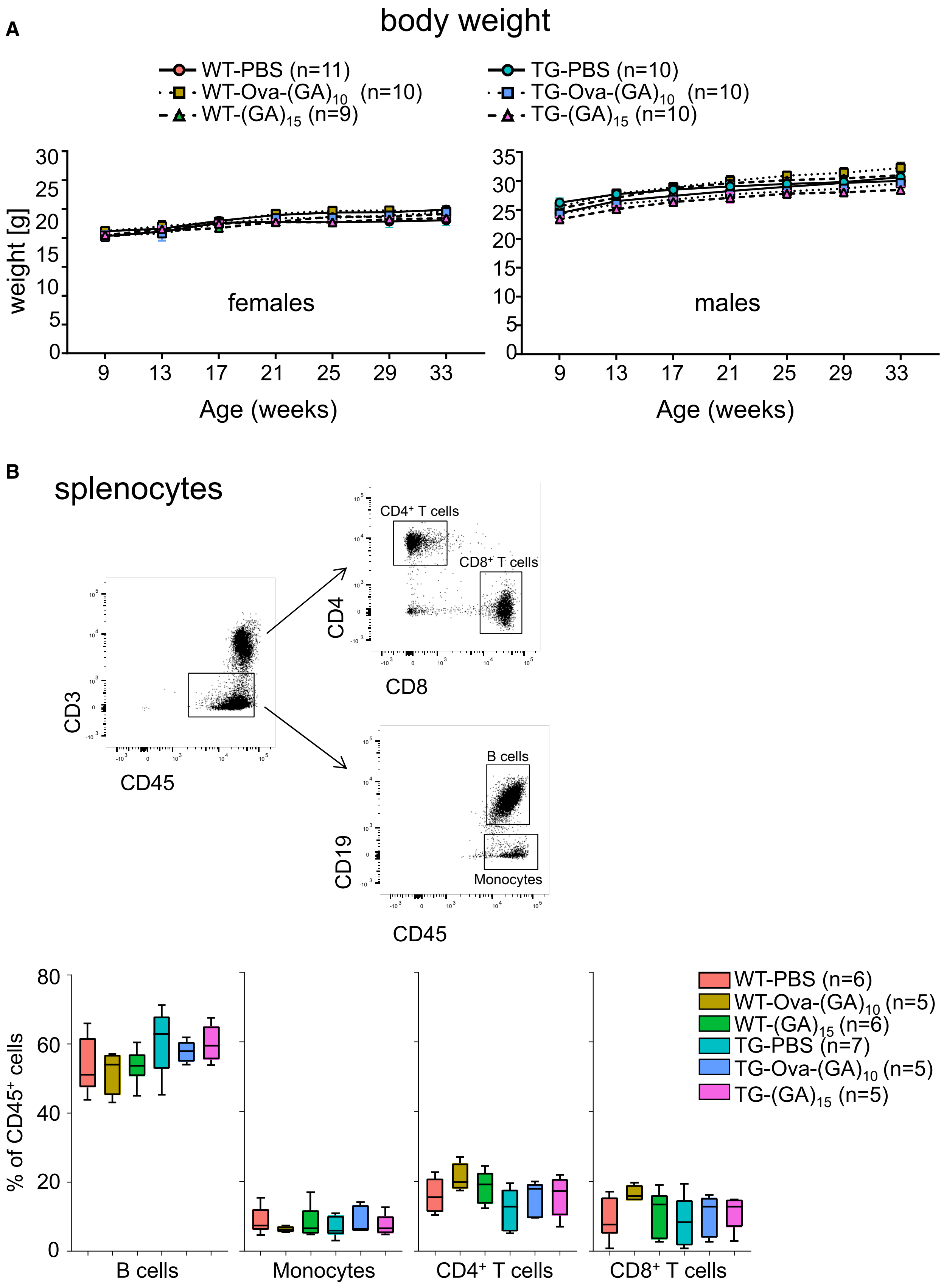
A
body weight
WT-PBS (n=11)
WT-Ova-(GA)10 (n=10)
WT-(GA)15 (n=9)
TG-PBS (n=10)
TG-Ova-(GA)10 (n=10)
TG-(GA)15 (n=10)
weight [g]
females
males
Age (weeks)
B
splenocytes
CD3
CD45
CD4
CD8
CD4+ T cells
CD8+ T cells
CD19
B cells
Monocytes
% of CD45+ cells
WT-PBS (n=6)
WT-Ova-(GA)10 (n=5)
WT-(GA)15 (n=6)
TG-PBS (n=7)
TG-Ova-(GA)10 (n=5)
TG-(GA)15 (n=5)
B cells
Monocytes
CD4+ T cells
CD8+ T cells

Figure EV1.

Figure EV2. Antisera detect poly-GA aggregates specifically.

A Immunoblot of HEK293 cells transfected with the indicated poly-GA-expressing construct and GFP control using monoclonal anti-GA clone 1A12 and antiserum from Ova-$(GA)_{10}$-vaccinated mice. Calnexin is used as a loading control. A representative of three experiments is shown.

B Immunoblot of HEK293 cells transfected with the indicated DPR-expressing construct and GFP control using monoclonal anti-GA clone 1A12 and antiserum from Ova-$(GA)_{10}$-vaccinated mice. Calnexin is used as a loading control. A representative of three experiments is shown.

C HEK293 cells were transfected with $(GA)_{175}$-GFP or GFP control and analyzed by immunofluorescence using a mouse monoclonal anti-GA antibody or antisera from vaccinated mice. Scale bar indicates 40 μm. A representative of three experiments is shown.

 © 2019 The Authors

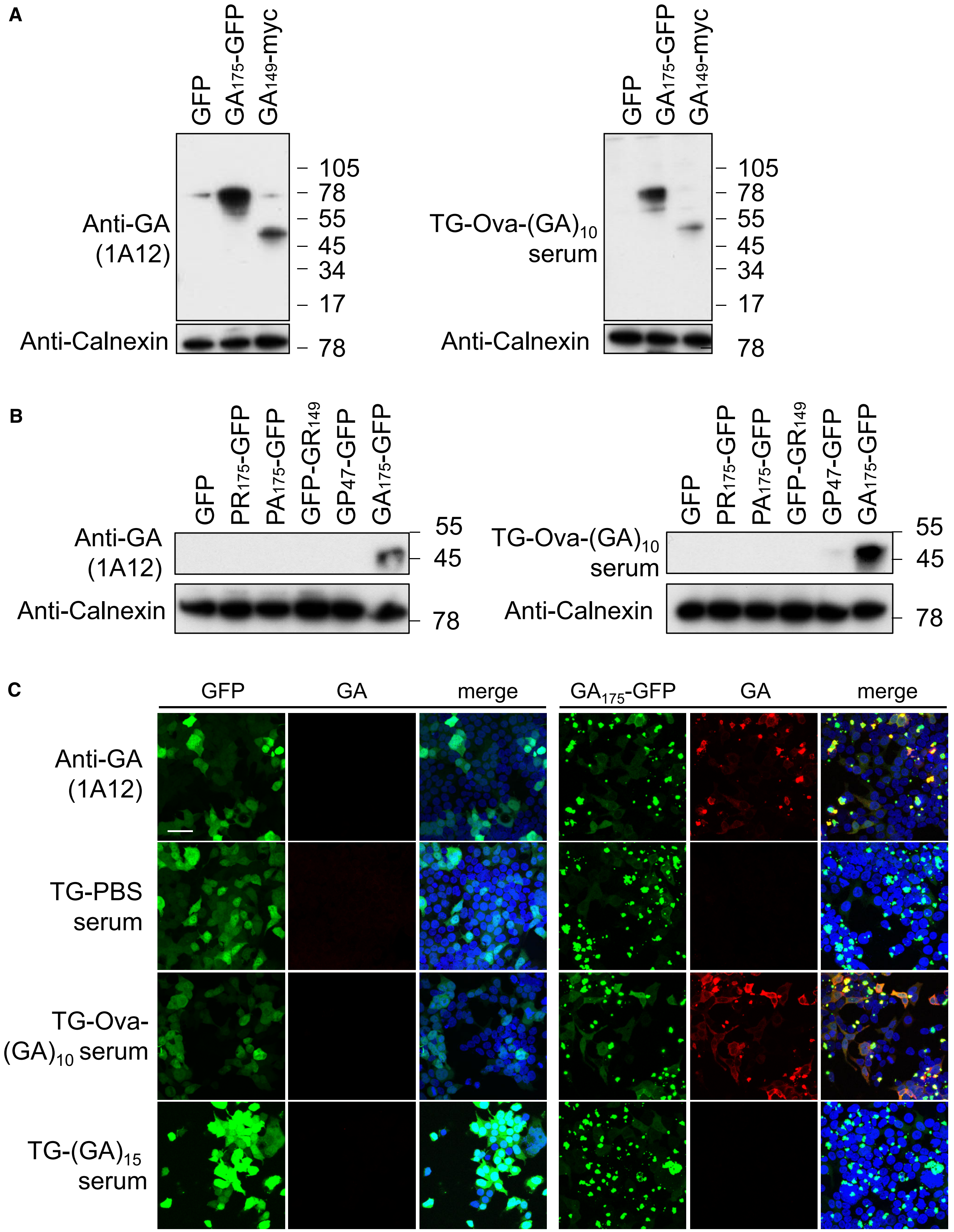
A
GFP
GA175-GFP
GA149-myc
Anti-GA (1A12)
TG-Ova-(GA)10 serum
105
78
55
45
34
17
Anti-Calnexin
78
B
GFP
PR175-GFP
PA175-GFP
GFP-GR149
GP47-GFP
GA175-GFP
Anti-GA (1A12)
TG-Ova-(GA)10 serum
55
45
Anti-Calnexin
78
C
GFP
GA
merge
GA175-GFP
Anti-GA (1A12)
TG-PBS serum
TG-Ova-(GA)10 serum
TG-(GA)15 serum

Figure EV2.

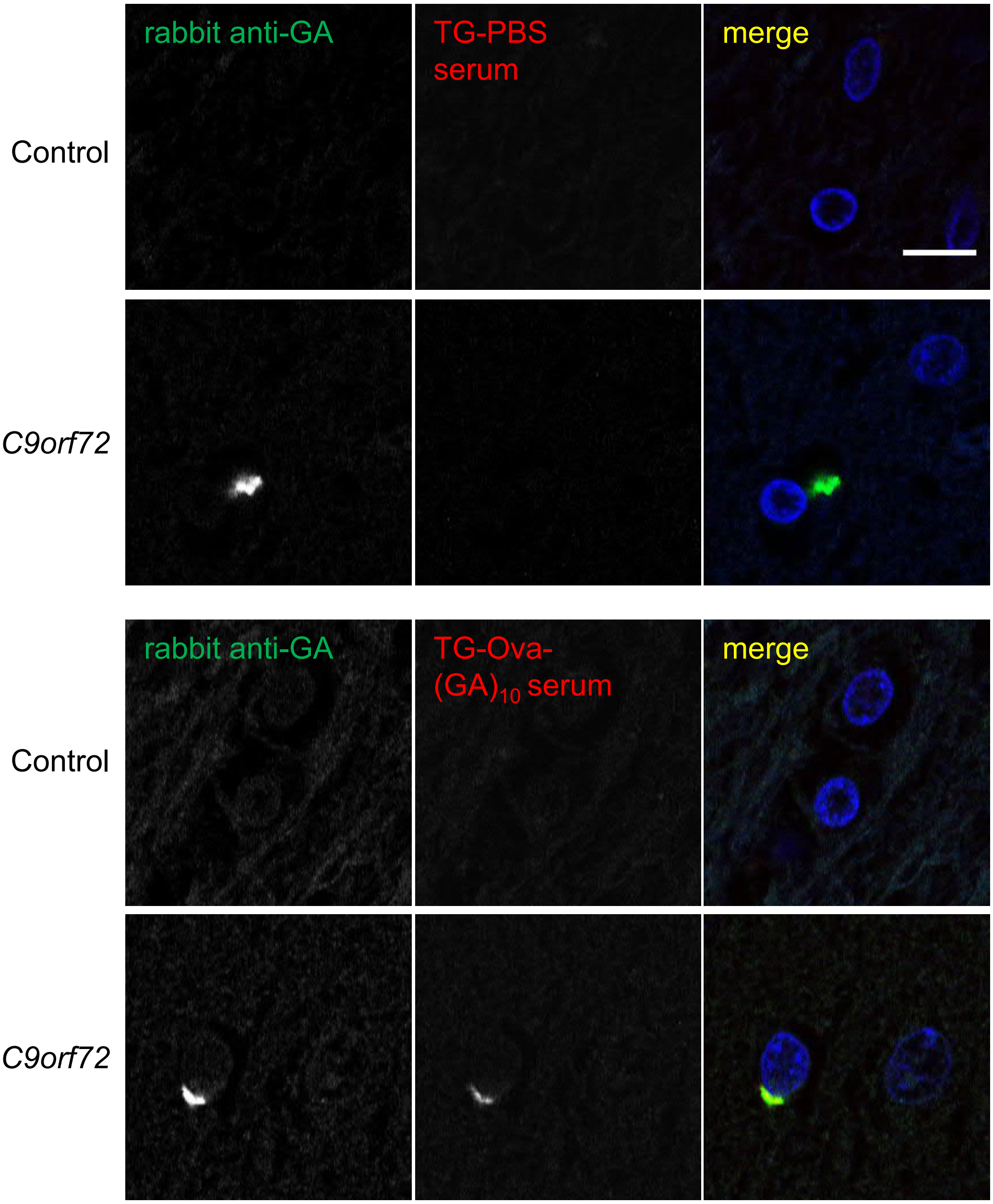

Figure EV3. Antisera detect poly-GA inclusions in *C9orf72* patients specifically.

Immunofluorescent staining of sections from a *C9orf72* patient and a healthy control. Neuronal cytoplasmic inclusions in occipital cortex were stained using a commercial rabbit polyclonal antibody (green) and antisera from Ova-GA-vaccinated mice (red). Nuclei were stained with DAPI (blue). Scale bar indicates 10 μm.

 © 2019 The Authors

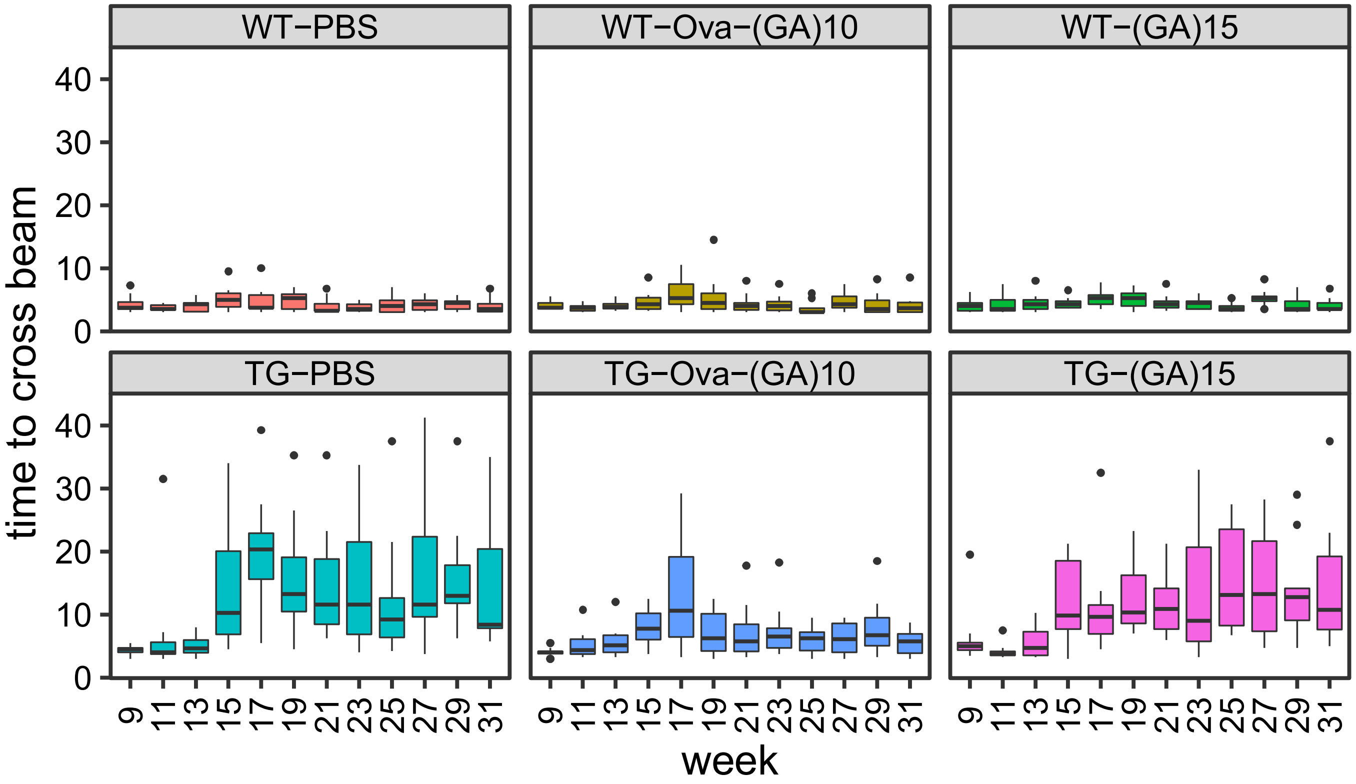

Figure EV4. Ova-GA immunization prevents motor deficits.

Longitudinal analysis of motor function in vaccinated GA-CFP mice and wild-type littermates in a beam walk assay (data from Fig 2A). n = 9–11 mice per group as indicated. Average time to cross the beam from duplicate repeat measurements in consecutive weeks. Tukey-style box plot shows 25th, 50th, and 75th percentiles, and whiskers extend to ± 1.5 interquartile range. Outliers depicted as dots. Statistics are shown in Fig 2A and Appendix Table S2.

Figure EV5. Ova-$(GA)_{10}$ immunization prevents microglia/macrophage activation and TDP-43 mislocalization.

A Analysis of microglia activation using Iba1 immunohistochemistry by measuring the area of Iba1 staining from complete spinal cord sections at 1-mm interval. Dot plot represents mean ± SD from n = 3 animals per group. One-way ANOVA, Tukey's *post hoc* test. ***$P < 0.001$, ns not significant. $F_{5,12} = 1.201$, $P = 0.3655$, TG-Ova-$(GA)_{10}$ vs. TG-PBS $P < 0.0001$, TG-$(GA)_{15}$ vs. TG-PBS $P = 0.8573$. TG-PBS vs. WT-PBS $P < 0.0001$.

B–D Automated analysis of microglia/macrophage morphology from 100-µm spinal cord sections stained for Iba1. Example reconstructions in (B). Scale bar indicates 30 µm. Colors in scatter plot indicate the different mice (3–4 mice per groups), blue area covers 25th to 75th percentiles and horizontal lines indicates 25th, 50th, and 75th percentiles respectively n = 35–95 number of microglia analyzed. Kruskal–Wallis test with Benjamini–Hochberg correction. **$P < 0.01$, ***$P < 0.001$. (C) Kruskal–Wallis $\chi^2 = 29.917$, df = 3, $P = 0.0000014$; TG-Ova-$(GA)_{10}$ vs. TG-PBS $P = 0.0003$; WT-PBS vs. TG-PBS $P = 0.000078$. (D) Kruskal–Wallis $\chi^2 = 28.532$, df = 3, $P = 0.0000028$; TG-Ova-$(GA)_{10}$ vs. TG-PBS $P = 0.00041$; WT-PBS vs. TG-PBS $P = 0.00032$.

E Representative immunofluorescence images of endogenous TDP-43 in the anterior horn of the spinal cord. In TG mice, more neurons show partial cytoplasmic mislocalization of TDP-43 (arrows). Scale bar indicates 20 µm. Analysis in Fig 3E.

© 2019 The Authors

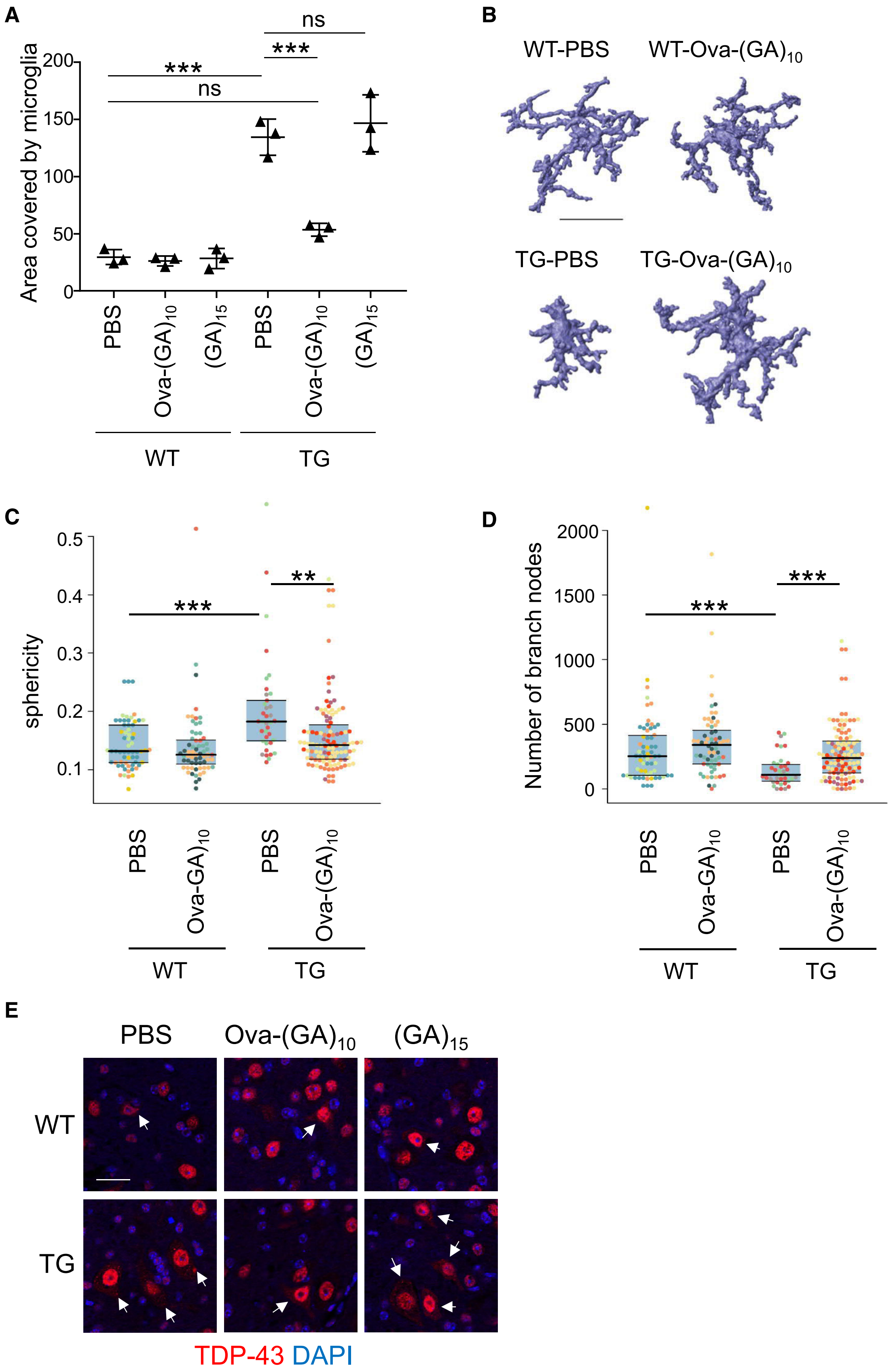

Figure EV5.

 © 2019 The Authors
